# Omics Technologies to Decipher Regulatory Networks in Granulocytic Cell Differentiation

**DOI:** 10.3390/biom11060907

**Published:** 2021-06-18

**Authors:** Svetlana Novikova, Olga Tikhonova, Leonid Kurbatov, Tatiana Farafonova, Igor Vakhrushev, Alexey Lupatov, Konstantin Yarygin, Victor Zgoda

**Affiliations:** Orekhovich Institute of Biomedical Chemistry, Pogodinskaya 10, 119121 Moscow, Russia; novikova.s.e3101@gmail.com (S.N.); ovt.facility@gmail.com (O.T.); kurbatovl@mail.ru (L.K.); farafonova.tatiana@gmail.com (T.F.); vakhrunya@gmail.com (I.V.); alupatov@inbox.ru (A.L.); kyarygin@yandex.ru (K.Y.)

**Keywords:** acute myeloid leukemia, HL-60 cell line, ATRA, induced differentiation, transcriptome, proteome, transcription factors, key molecules, regulatory pathway modelling, SRM

## Abstract

Induced granulocytic differentiation of human leukemic cells under all-*trans*-retinoid acid (ATRA) treatment underlies differentiation therapy of acute myeloid leukemia. Knowing the regulation of this process it is possible to identify potential targets for antileukemic drugs and develop novel approaches to differentiation therapy. In this study, we have performed transcriptomic and proteomic profiling to reveal up- and down-regulated transcripts and proteins during time-course experiments. Using data on differentially expressed transcripts and proteins we have applied upstream regulator search and obtained transcriptome- and proteome-based regulatory networks of induced granulocytic differentiation that cover both up-regulated (HIC1, NFKBIA, and CASP9) and down-regulated (PARP1, VDR, and RXRA) elements. To verify the designed network we measured HIC1 and PARP1 protein abundance during granulocytic differentiation by selected reaction monitoring (SRM) using stable isotopically labeled peptide standards. We also revealed that transcription factor CEBPB and LYN kinase were involved in differentiation onset, and evaluated their protein levels by SRM technique. Obtained results indicate that the omics data reflect involvement of the DNA repair system and the MAPK kinase cascade as well as show the balance between the processes of the cell survival and apoptosis in a p53-independent manner. The differentially expressed transcripts and proteins, predicted transcriptional factors, and key molecules such as HIC1, CEBPB, LYN, and PARP1 may be considered as potential targets for differentiation therapy of acute myeloid leukemia.

## 1. Introduction

Cell differentiation is a fundamental process of the development, growth, reproduction of multicellular organisms. Regulation of cell differentiation has been for decades and remains an important task for investigation due to its importance in cancer and many other diseases therapy. Leukemic cells that are induced to differentiate under all-*trans*-retinoid acid (ATRA) treatment make a convenient model for studying of cell maturation in vitro.

Normally, ATRA in physiological dosage binds and activates a heterodimer receptor RAR/RXR followed by release of histone deacetylases (HDACs) and transcription co-repressors (N-CoR or SMRT), and by recruitment of transcription co-activators (NcoA-1/SRC-1, CBP/p300, p/CIP, and ACTR) [1]. In turn, retinoic acid response element (RARE) containing genes, which are repressed by nonactive RAR/RXR, trigger the further cascade of molecular events leading to myeloid precursor’s maturation into functional granulocytes. Various mutations impair granulocytic differentiation resulting in highly heterogeneous acute myeloid leukemia (AML), which could be cured by high dosage of ATRA. In the case of AML subtype M3 (French–American–British (FAB) classification), a.k.a acute promyelocytic leukemia (APL), deleterious mutation, namely balanced chromosomal translocation between chromosomes 15 and 17 t (15;17) (q24; q21), affects retinoic acid (RA) receptor gene *RARα* resulting in formation of dominant negative fusion protein PLM-RARA [2]. The NB4 cell line that harbors such a hallmark mutation, is used as a model to study of the APL cell biology [3]. In APL cells the transcription of RA-responsive genes is blocked due to the increased avidity of PLM-RARA/RXR for co-repressor molecules [4]. The treatment with high dosage of ATRA induces dissociation of co-repressors from PML-RARA and triggers fusion protein degradation via the ubiquitin-proteasome or autophagy pathway [5,6]. The ATRA-based regimens that are used as a first-line treatment of APL patients, induce complete remission rates of 90% [7]. Nevertheless, other types of AML are not that successfully treatable with the 5-years survival rates only about 40–45% [8]. Meanwhile, antileukemic effect of ATRA was also observed in AML (non-APL) cell models, including HL-60, THP-1, MOLM-14, HF-6, and U937 cell lines [9].

The HL-60 promyelocytic leukemia cell line is classified as AML with maturation, also referred to as AML subtype M2 by FAB classification [10]. These cells were isolated in 1977 from a patient with acute myeloid leukemia. Later it was found that, these promyelocytic cells could be induced to differentiate into granulocytes in vitro by ATRA [11]. The HL60 cell genome contains normal *RARα* gene, an amplified *c-myc* proto-oncogene and deficient of *p53* gene [12,13]. Notably, deletion in the *p53* gene occurs at a frequency of up to 10% in de novo AML (non-APL) cases and associated with exceedingly adverse prognosis regardless of the type of mutation (missense, nonsense, small insertions, and deletions, etc.) [8]. Being ATRA-responsive, the HL60 cell line has been used for decades as a convenient model object for cell differentiation [11,14].

Omics technologies represent powerful tools for a full-scale analysis of gene and protein expression that allow for gaining important molecular information about differentiation process, and acquiring the complete picture of the cell maturation. Thus, using HL-60 (AML) and NB4 (APL) cell lines as model systems, the complexity of differentiation processes and the diversity of pathways involved in induced differentiation at transcriptome [15,16,17] and proteome [18,19] levels have been demonstrated.

Despite the fact that proteomics and transcriptomics alone represent the powerful techniques for investigation of ATRA-induced differentiation, the systems approach is appealing to the elucidation of molecular mechanisms. In this respect, the systems study was performed on the NB4 promyelocytic cell line under ATRA treatment (alone or in combination with arsenic trioxide (ATO)) in a time-course manner. By applying microarray technology and 2D-gel electrophoresis followed by MALDI-TOF-TOF analysis, transcription factors (TFs) and co-factors responsible for global changes in transcriptional regulation and involved in stimulation of the IFN-pathway, cell cycle arrest, and activation of signal transduction have been unmasked [3].

However, even simultaneous analysis of proteome and transcriptome differences observed in the experiment is not always sufficient to unravel regulatory mechanisms. The up- or down-regulation of protein and transcript levels under ATRA treatment is often caused by previous regulatory events. Predicting transcription factors, responsible for altered gene expression, and revealing, in turn, their putative regulators, a hierarchical model of induced differentiation could be built. Therefore, a bioinformatics search for upstream regulators, including transcription factors [20], is an appropriate tool for proteome and transcriptome data interpretation. Identification and analysis of TFs and regulatory pathways responsible for altered gene or protein expression that result in the cell differentiation may contribute to identification of the mechanism(s) underlying this complex process.

## 2. Materials and Methods

### 2.1. Experimental Design

The time-course studying of induced granulocytic differentiation allows obtainment of the most accurate data on molecular perturbations under ATRA treatment. Previously, several schedules of HL-60 cell harvesting after ATRA treatment have been applied in the time-course experiments [3,21]. To perform transcriptomic and proteomic profiling, we selected 24 and 96 h time points, when the molecular perturbations are prominent. To reveal the molecular onset of cell maturation at transcriptome and proteome levels, we also added the 3 h time point. For proteomic experiment we also studied the time point 48 h of treatment; during this period HL-60 cells underwent two division cycles. In our preliminary mass-spectrometry experiments we did not observe any significant changes in the ATRA-induced cell proteome within the first 2 h (compared to 0 h) after ATRA induction or at 72 h (compared to 96 h) after treatment (data not shown).

For proteome analysis, we performed the ATRA-induced differentiation experiments in three independent biological replicates. HL-60 cells were harvested at 0, 3, 24, 48, and 96 h after ATRA treatment (overall 15 samples). For the transcriptome analysis, HL-60 cells were subjected to ATRA treatment in three biological replicates and were harvested at 0, 3, 24, and 96 h (overall 12 samples).

For the proteome analysis, the LC-MS/MS experiments were carried out in five technical replicates per time point, and the whole-genome transcriptome analysis was performed in three technical replicates per time point.

**Figure 1:**
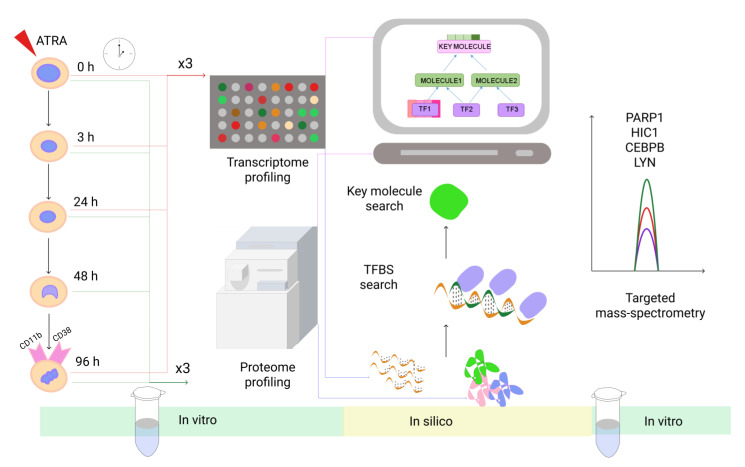
The study workflow. We applied a multi-disciplinary platform to study ATRA-induced granulocytic differentiation in a time-course manner using HL-60 cell line as a model. We combined LC-MS/MS analysis (0, 3, 24, 48, and 96 h after ATRA treatment, three bio repeats), whole-genome transcriptome analysis (0, 3, 24, and 96 h after ATRA treatment, three bio repeats), and bioinformatic search for transcription factor binding sites (TFBS) and for the key regulatory molecules. To verify the predicted regulatory networks the abundance of proteins HIC1, CEBPB, LYN, and PARP1, belonging to the designed model regulatory networks or involving in differentiation onset, were measured in time-course manner by selected reaction monitoring (SRM) using synthetic isotopically-labeled peptides as standard.

Cells harvested before ATRA treatment (time point 0 h) served as controls for both transcriptomic and proteomic profiling. The study workflow is shown in Figure 1.

### 2.2. HL60 Cells Cultures

The HL-60 human promyelocytic leukemia cells (obtained from the cell culture bank Institute of Biomedical Chemistry (IBMC), Moscow, Russia) were grown in RPMI-1640 medium supplemented with 10% fetal bovine serum, 100 U/mL penicillin, 100 U/mL streptomycin and 2 mM L-glutamine (all Gibco™, Paisley, UK) in a CO_2_ incubator under standard conditions (37 °C, 5% CO_2_, 80% humidity). ATRA (Sigma-Aldrich, St. Louis, MO, USA) was dissolved in ethanol as a stock solution at 1 mM. HL-60 cells were treated with ATRA as described in [3] and control HL-60 cells were treated with an equal volume of the solvent (ethanol).

Cell differentiation was evaluated by the CD11b and CD38 expression measured by flow cytometry. At the selected time points, the cells were harvested, washed twice with PBS, transferred to 1.5-mL Eppendorf tubes, and pelleted by centrifugation at 3000× *g* for 15 min using an Eppendorf 5424R centrifuge (Eppendorf, Hamburg, Germany). After removing the supernatants, the cell pellets were frozen in liquid nitrogen and stored until transcriptomic and proteomic analysis.

### 2.3. Transcriptome Analysis

Total RNA was isolated from the cells using RNeasy Mini Kit (Qiagen, Hilden, Germany) at each time point studied. The quality of the extracted RNA was controlled using a Bioanalyzer 2100, RNA 6000 Nano LabChips, and the 2100 Expert standard software (all Agilent Technologies, Santa Clara, CA, USA). Approximately 0.5 µg of each RNA sample was used for cDNA preparation in the reaction of the reverse transcription performed using a Low RNA Input Linear Amp Kit (Agilent Technologies, Santa Clara, CA, USA) according to standard protocol. The cRNA samples for all time points were labeled with Cy5-CTP (Perkin Elmer, Waltham, MA, USA) and with Cy3-CTP (Perkin Elmer, Waltham, MA, USA) for the control sample (the time point 0 h). The cRNA fragmentations and hybridizations were performed using a standard protocol with an in situ Hybridization Kit Plus (Agilent Technologies, Santa Clara, CA, USA). Data acquisition was carried out using a DNA Microarray Scanner G2505C (Agilent Technologies, Santa Clara, CA, USA). The primary transcriptome data were processed using the Feature Extraction software (version 10.1.3.1; Agilent Technologies, Santa Clara, CA, USA).

Statistical data analysis by ANOVA with the *p*-value cut-off set at 0.05 was performed using the GeneSpring GX12.5 software (Agilent Technologies, Santa Clara, CA, USA). Thus, we prepared the lists of genes that showed more than two-fold expression difference at least at one time point studied.

### 2.4. Preparation of HL60 Cells Lysates and In-Solution Digestion with Trypsin

The cell samples were lysed using ice-cold buffer (150 µL) containing 3% sodium deoxycholate, 2.5 mM EDTA, 75 mM Tris-HCl (all Sigma-Aldrich, St. Louis, MO, USA), pH 8.5 and protease inhibitors cOmplete™ (Roche, Basel, Switzerland) with subsequent ultrasonication using the Bandelin Sonopuls probe (“BANDELIN electronic GmbH & Co. KG”, Berlin, Germany). The cell lysates were centrifuged for 15 min at 5000× *g* using Eppendorf 5424R centrifuge. The supernatants were collected, and the pellets were dissolved in 100 µL of lysis buffer, and then subjected to the second round of protein solubilization as described above. The sample protein concentration was measured using a Pierce™ BCA Protein Assay Kit (Pierce, Rockford, IL, USA). Protein digestion was performed according to the protocol described in detail by Zgoda et al. [22]. Briefly, the protein sample (about 100 µg) was transferred into a clean tube and denaturation solution (5 M urea, 1% sodium deoxycholate, in a 50 mM triethylammonium bicarbonate buffer (TEAB) containing 20mM dithiothreitol (DTT) (all Sigma-Aldrich, St. Louis, MO, USA) 20 mM DTT) in volume of 20 µL was added to make the final concentration of total protein close to 5 mg/mL. Then the samples were heated for 60 min at 42 °C and, after cooling at room temperature, 25 µL of 15 mM 2-iodoacetamide in 50 mM TEAB was added. The alkylation reaction continued for 30 min at room temperature and the sample was then diluted up to 120 µL by 50 mM TEAB to decrease the final concentration of denaturation buffer compounds and dilute the final protein concentration close to 0.5 mg/mL. Trypsin (1 µg) was added to samples and incubated overnight at 37 °C. The hydrolysis was stopped by adding formic acid (to a final concentration of 5%). Samples were centrifuged for 10 min at 10 °C at 12,000× *g* to sediment deoxycholic acid. The supernatant was transferred into a clean tube. In the obtained supernatants, the total peptide concentration was determined by the colorimetric method using a Pierce™ Quantitative Colorimetric Peptide Assay kit (Thermo Scientific, Waltham, MA, USA) in accordance with the manufacturer’s recommendations. The peptides were dried and dissolved in 0.1% formic acid to a final concentration of 1 µg/µL.

### 2.5. Shotgun Mass Spectrometry

The peptide samples obtained were analyzed using the Agilent HPLC system 1100 Series (Agilent Technologies, Santa Clara, CA, USA) connected to a hybrid linear ion trap LTQ Orbitrap Velos, equipped with a nanoelectrospray ion source (Thermo Scientific, Waltham, MA, USA). Peptide separations were carried out on a RP-HPLC Zorbax 300SB-C18 column (C18 3.5 µm, 75 µm inner diameter and 150 mm length, Agilent Technologies, Santa Clara, CA, USA) using a linear gradient from 95% solvent A (water, 0.1% formic acid) and 5% solvent B (water, 0.1% formic acid, and 80% acetonitrile) to 60% solvent B over 85 min at a flow rate of 0.3 µL/min.

Mass spectra were acquired in the positive ion mode using Orbitrap analyzer with a resolution of 30,000 (*m/z* = 400) for MS and 7500 (*m/z* = 400) for MS/MS scans. The AGC target was set at 2 × 10^5^ and 1 × 10^5^ with maximum ion injection time 50 ms and 100 ms for MS and MS/MS, respectively. Survey MS scan was followed by MS/MS spectra for five the most abundant precursors. The higher energy collisional dissociation (HCD) was used, and normalized collision energy was set to 35 eV. Signal threshold was set to 5000 for an isolation window of 2 *m/z*. The precursors fragmented were dynamically excluded from targeting with repeat count 1, repeat duration 10 s, and exclusion duration 60 s. Singly charged ions and those with not defined charge state were excluded from triggering the MS/MS scans.

### 2.6. Data Analysis

The mass spectrometry data were analyzed using SPIRE pipeline [23]. The raw mass spectrometry data were converted to the mzXML format with the RawToMzXML convertor and uploaded into the SPIRE server. The experimental data were assigned to five time points (0, 3, 24, 48, and 96 h); each point included three biological- with five technical replicates. The data obtained were searched by the in-built «Composite» search engine within SPIRE pipeline using the following parameters: enzyme specificity was set to trypsin, two missed cleavages were allowed. Carbamidomethylation of cysteines was set as fixed modification and methionine oxidation was set as variable modification for the peptide search. The mass tolerance for precursor ions was 10 ppm; the mass tolerance for fragment ions was 20 ppm. Human FASTA file (September 2015) was used as a protein sequence database. The spectra identified with 90% probability were assigned to peptides. The local false discovery rate for protein identification was set bellow 0.01 (locFDR < 0.01). locFDR was calculated in SPIRE utilizing randomized or decoy database searches [23].

Label-free quantitation was performed with the use of the SPIRE software by default settings. Expression ratios and *p*-values were calculated based on an over-dispersed Poisson model using an empirical Bayes correction [23]. The proteins with the expression fold change > 1.5, *p*-value < 0.05 and CV between biological repeats < 30%, were considered as differentially expressed. The imputation of missing data has not been applied to mass-spectrometric results.

The volcano plot was obtained using VolcaNoseR web app [24].

### 2.7. Functional Classification of Differentially Expressed Genes and Proteins

Functional analysis of differentially expressed genes/proteins was carried out using the «Functional classification» option of the geneXplain platform (http://platform.genexplain.com) with GO and PROTEOME Databases (BIOBASE) implemented as a module of the GeneXplain platform.

For the functional analysis of gene groups exhibiting altered expression at the selected time points of cell differentiation, the cut-off value for the probability of random gene allocation of a gene to a particular group (Adjusted *p*-value) was set at 5 × 10^−4^. Only statistically significant classification of genes according to the GO categories, describing various biological processes in cells, was taken into consideration for the functional analysis.

The STRING database v.11.0 was used to retrieve the protein–protein interactions (PPIs) from the lists of DEGs of MCD group at 3, 24, and 96 h. A high confidence (0.9) score was applied. The active interaction sources were experiments and curated databases. The built-in functional enrichment analysis results according to the molecular function (GO), and KEGG pathways were used for visualization.

### 2.8. Search for Transcription Factors, Putatively Regulating Gene and Protein Expression during ATRA-Induced Differentiation of HL-60 Cells

The search for over-represented transcription factor binding sites (TFBS) was performed using geneXplain platform 2.0 software packages (http://platform.genexplain.com) and TRANSFAC^®^ database [25]. The differentially expressed genes/proteins at different time points were considered as the test sets (Yes-sets). The gene/protein that did not show any expression changes after ATRA treatment were used as a background set (No-sets). The profile used for analysis contains a collection of vertebrate non-redundant transcription factor matrices. The promoter window was selected from −1000 to +100 from the transcription start site, and only the best-supported promoters of the genes analyzed were used. The cut-off values with a threshold of *p*-value < 0.005 were selected to obtain high-scoring binding sites. The matrices with high over-representation of site frequency in the promoters under study versus the background promoters (ratio > 1.4) were selected for further analysis. These matrices were converted to the set of the transcription factors (TFs), which can be responsible for expression changes in the group of genes/proteins under study.

### 2.9. Generation of Regulatory Networks

The identification of potential master regulators in the signal transduction network was performed using the «Regulator search» module of the geneXplain platform 2.0 software (http://platform.genexplain.com). The signal transduction network was provided by the manually curated database, TRANSPATH^®^. The algorithm starts from a set of TFs and performs a graph-topological search in the signal transduction network upstream of transcription factors to identify the “key nodes” that can play a crucial role in intracellular signaling from various receptors to the set of TFs identified. These key nodes may be considered as master regulators of the process studied. The following setting parameters were used: TRANSPATH^®^ database, maximal search radius R = 10, Score cutoff = 0.2, FDR cutoff = 0.05 and Z-score cutoff = 1.0. Besides FDR, for each possible additional regulator the Score, Z-score and Ranks sum values were calculated. For the proteomic data analysis, the “Context genes” option was used for the search of key regulators. In this case, passing through the common network nodes, the nodes presented at the transcriptome data were preferentially selected. Among the overall list of regulators generated after the search, the statistically significant results were selected using the Ranks sum parameter. Thus, it was possible to find the molecules characterized by equally good “Score” and “Z-score” parameters. The “Score” parameter reflects how well a key molecule is associated with the other molecules in the database and how many molecules of the input TFs are present in the network for a given key molecule. The “Z-score” reflects how the proposed molecule corresponds to the input TFs set. The ranks sum is a combination of Score and Z-score. In other words, these “trivial” expected results attract interest as the well-known “nodes” in the network (Score) and more specific key molecules for the input sample, which are less likely to be detected as an important regulator in the case of the other TF sets used simultaneously.

### 2.10. Selected Reaction Monitoring (SRM)

The standard peptides for HIC1 (LEEAAPPSDPFR), CEBPB (VLELTAENER) LYN (TQPVPESQLLPGQR), and PARP1 (TLGDFAAEYAK) were obtained using the solid-phase peptide synthesis on the Overture™ Robotic Peptide Library Synthesizer (Protein Technologies, Manchester, UK) or Hamilton Microlab STAR devices according to the published method [26]. The isotopically labeled lysine (^13^C_6_,^15^N_2_), arginine (^13^C_6_,^15^N_4_) or serine (^13^C_3_,^15^N_1_) leucine (^13^C_6_,^15^N_1_) were used for isotopically labeled peptide synthesis instead of the unlabeled lysine (TLGDFAAEYA**K**), arginine (VLELTAENE**R**), leucine (TQPVPESQL**L**PGQR), or serine (LEEAAPP**S**DPFR), respectively. Concentrations of the synthesized peptides were measured by the method of amino acids analysis with fluorescent signal detection of amino acids derived after acidic hydrolysis of peptides as described in [27].

SRM experiments were performed in three biological replicates with five time points each (0, 3 h, 24 h, 48 h, and 96 h) and in five technical replicates for each time point studied. The digested samples were spiked with isotopically labeled peptide to the final concentration 50 fmol/µg of total protein. Peptide samples (2 µg) were separated on a RP-C18 column, (Zorbax 300SB-C18, 3.5 m, 150 mm × 0.075 mm, Agilent Technologies, Santa Clara, CA, USA) using the nanoflow UPLC DionexUltiMate 3000 RSLC nano System Series (Thermo Scientific, Waltham, MA, USA). Peptide separation was achieved using a linear gradient from 95% solvent A (0.1% formic acid) and 5% solvent B (80% acetonitrile, 0.1% formic acid) to 60% solvent A and 40% solvent B over 25 min at a flow rate of 0.4 µL/min. SRM analysis was performed on the QqQ TSQ Vantage (Thermo Scientific, Waltham, MA, USA) with capillary voltage set at 2100 V, isolation window was set to 0.7 Da. SRM transition details for all peptides are shown in Table S8. The results were processed using Skyline software v4.1.0 (MacCoss Lab Software, Seattle, WA, USA). The coefficient of variation (CV) of transition intensity did not exceed 25%, 12%, 12%, and 6% between technical replicates for LEEAAPPSDPFR, VLELTAENER TQPVPESQLLPGQR, and TLGDFAAEYAK, respectively.

## 3. Results

### 3.1. Transcriptome Analysis and Functional Annotation of Differentially Expressed Genes during ATRA-Induced Differentiation of HL-60 Cells

To validate HL-60 cell differentiation into neutrophils, expression of surface markers CD11b and CD38 was assessed by flow cytofluorometry at 96 h after ATRA treatment prior transcriptome/proteome analysis (Figure S1). Although measurement of CD11b is the most convenient way to evaluate granulocyte differentiation, to obtain more accurate data we have used additional marker CD38 that promotes induced myeloid maturation [28]. The mean fluorescence from HL-60 cells at 96 h after ATRA-treatment increased approximately 15-fold (CD38-from 171 to 2929; CD11b-from 112 to 1726) compared to untreated control. This indicates that the granulocyte differentiation of the HL60 cell line was successful.

To obtain the transcriptomic data, HL-60 cells were harvested at 3 h, 24 h, and 96 h after ATRA treatment followed by mRNA microarray profiling. A total of 14,543 gene expressions were detected at all the time points studied. Among them 159, 231, and 1449 genes with fold-change (FC) ≥2 were determined as differentially expressed genes (DEGs) at 3 h, 24 h, and 96 h after ATRA treatment, respectively (Supplemental Table S1).

**Figure 2:**
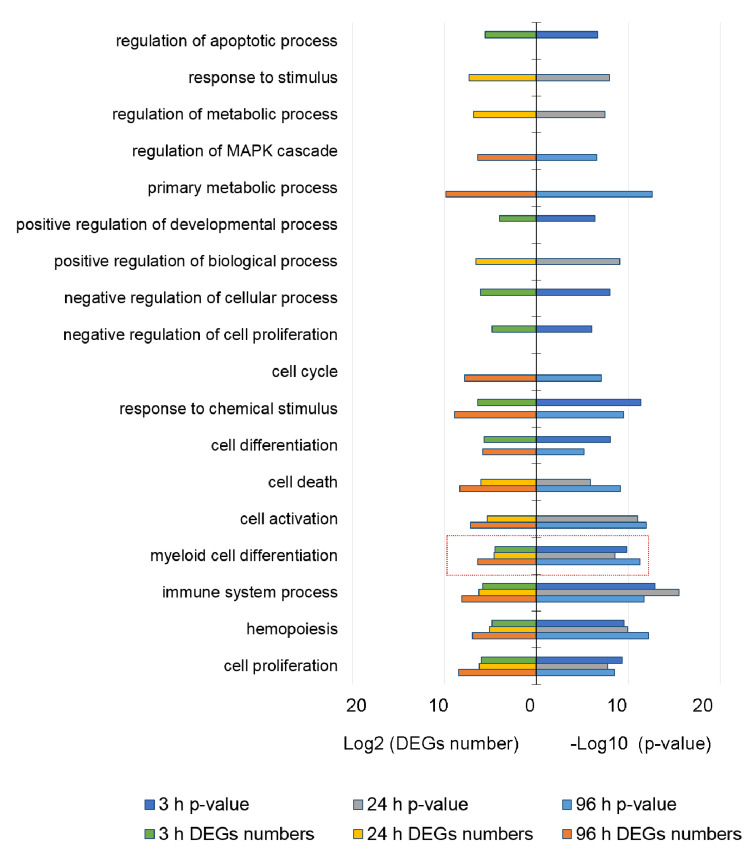
The functional GO analysis of differentially expressed genes (DEGs) of HL-60 cells at 3 h, 24 h, and 96 h after ATRA treatment. The number of DEGs (Log2 transformed) and *p*-value (-Log10 transformed) are provided on the x-axis. The groups from the category of “Biological process” are on the y-axis. The threshold adjusted *p*-value < 10^−4^. The group of “myeloid cell differentiation” (MCD, GO: 0030099) is marked by red color.

Further, we focused on the bioinformatics reconstruction of putative regulatory pathways for DEGs that were involved in cell differentiation according to highly validated data. We annotated the altered expression genes by the Gene Ontology (GO) database category related to the biological processes (Figure 2).

Figure 2 shows the DEGs at all time points were enriched by molecules, which were assigned to the group of “myeloid cell differentiation” (MCD, GO: 0030099). The MCD group was revealed at 3 h after ATRA treatment with 22 DEGs, and then was expanded up to 24 and 81 DEGs at 24 h, and 96 h, respectively.

The results of the interaction analysis by STRING (Figure S2) show that the DEGs of MCD group were enriched in their interaction with the highest confidence (0.9). The KEGG database annotation revealed mapping of the DEGs of MCD group into “Chemokine signaling pathway” at 3 h and 24 h, and into “NOD-like receptor signaling pathway” at 96 h.

While the data for 3 h and 24 h suggest the cytokine signaling as one of the mechanisms of the ATRA-induced granulocytic differentiation, the results for 96 h indicate the manifestation of functions of already mature neutrophils. These observations emphasize that the bioinformatics mapping of molecules with altered expression on known signaling pathways is insufficient for a complete understanding of the regulatory events.

Moreover, the earliest time point (3 h after ATRA treatment) provides transcriptomic data on the granulocytic differentiation onset. The DEGs of the MCD group at 3 h included ASB2, BCL2A1, CCL2, CCL3L1, CCL4, CCR5, CD300A, CD38, CEBPB, FGR, HES1, HNRPLL, IL8, LRG1, LYN, RELB, TNFAIP2, BCL11A, NR2F2, PTGER2, RGS18, and SERPINB2. Among them CEBPB, CCR5, CCL4, FGR, CXCL8 (IL8), and LYN form a putative functional complex according to the STRING interaction analysis (Figure S2a). These data are of great importance for deciphering the very first molecular events of ATRA-induced granulocytic differentiation. Further, the dynamics of transcription factor CEBPB and LYN kinase was assessed by targeted mass-spectrometry approach (selected reaction monitoring (SRM)) at protein level.

The MCD group genes have been used for following upstream regulators search. The lists of the MCD group genes are presented in Supplemental Table S2.

### 3.2. Proteomic Analysis and Functional Annotation of Differentially Expressed Proteins during ATRA-Induced Differentiation of HL-60 Cells

Proteome dynamics is associated with cell phenotype development and its continuous observation can contribute to understanding of the cell maturation process. Previously, for systems analysis of induced granulocyte differentiation and apoptosis under ATRA/arsenic trioxide treatment starting time points of 6 h at transcriptomic level and 12 h at proteomic level were used [3]. We tried to unveil the molecular onset of differentiation. In our preliminary experiments we did not observed any significant changes in the ATRA induced cell proteome within the first 2 h after ATRA induction (data not shown). We performed proteomic profiling of HL-60 cells at 0, 3 h, 24 h, 48 h, and 96 h after ATRA-treatment.

Using “Composite” search engine in the SPIRE software, we identified 1436, 1470, 1379, 1253, and 1210 proteins with (locFDR) < 0.01 at the 0, 3 h, 24 h, 48 h, and 96 h time points, respectively (Supplemental Tables S3 and S4). Mass-spectrometric data are available via the ProteomeXchange with identifier PXD006768. Based on label free quantitative analysis, 122, 169, 199, and 275 proteins were revealed as differentially expressed proteins (DEPs) (FC ≥ 1.5, *p*-value < 0.05, CV < 30%) at 3, 24, 48, and 96 h after ATRA treatment comparing to control (0 h), respectively. Data on label free quantitative analysis and relative expression are presented in Supplemental Table S5. The heatmap of protein expression is presented in Figure S3. The DEPs are listed in Table S5.

**Figure 3:**
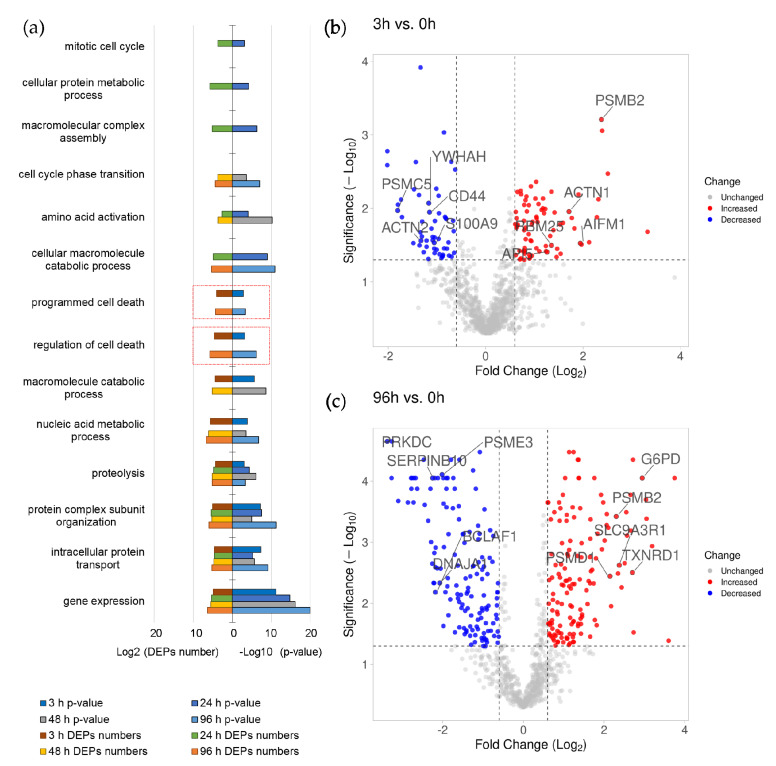
(**a**) The functional GO analysis of differentially expressed proteins (DEPs) of HL-60 cells at 3 h, 24 h, 48 h, and 96 h after ATRA treatment. The number of DEPs (Log2 transformed) and *p*-value (-Log10 transformed) are provided on the x-axis. The groups from the category of “Biological process” are on the y-axis. The threshold adjusted *p*-value < 10^−4^. The groups containing proteins regulating cell death and apoptosis are marked by red. The volcano plots show the differences in proteins abundance at 3 h (**b**) and 96 (**c**) after ATRA treatment; significantly up- and down-regulated proteins are shown as red and blue dots, respectively; names are shown for five most up- and down-regulated proteins that were annotated by GO belonging to groups “programmed cell death” and/or “regulation of cell death”.

The functional analysis of DEPs was performed in the same way as for the DEGs. The results are shown in Figure 3.

Figure 3a shows that the DEPs are enriched with the proteins involved in programmed cell death and its regulation at 3 h and 96 h after ATRA treatment. The five most up-regulated DEPs involved in programmed cell death at 3 h after ATRA-treatment comprise proteasome subunit beta type-2 (PSMB2, P49721), apoptosis-inducing factor 1 (AIFM1, O95831), alpha-actinin-1 (ACTN1, P12814), RNA-binding protein 25 (RBM25, P49756), and apoptosis inhibitor 5 (API5, Q9BZZ5). The top five down-regulated DEPs included 26S proteasome regulatory subunit 8 (PSMC5, P62195), alpha-actinin-2 (ACTN2, P35609), 14-3-3 protein eta (YWHAH, Q04917), CD44 antigen (CD44, P16070), and protein S100-A9 (S100A9, P06702).

The five most up-regulated proteins at 96 h after ATRA-treatment included 26S proteasome non-ATPase regulatory subunit (PSMD1, Q99460), proteasome subunit beta type-2 (PSMB2, P49721), glucose-6-phosphate 1-dehydrogenase (G6PD, P11413), thioredoxin reductase 1 (TXNRD1, Q16881), and Na(+)/H(+) exchange regulatory cofactor NHE-RF1 (SLC9A3R1, O14745). Although these DEPs are assigned to the groups regulating cell death, they affect cell fate indirectly through metabolic effects. The 5 most down-regulated DEPs included DNA-dependent protein kinase catalytic subunit (PRKDC, P78527), Bcl-2-associated transcription factor 1 (BCLAF1, Q9NYF8), DnaJ homolog sub-family A member 1 (DNAJA1, P31689), proteasome activator complex subunit 3 (PSME3, P61289), and serpin B10 (SERPINB10, P48595).

The STRING interaction analysis (Figure S4) revealed that the DEPs of group “programmed cell death” and/or “regulation of cell death” were enriched in their interaction with the highest confidence (0.9) at 3 h and 96 h after ATRA-treatment. Moreover, these proteins were mapped to the “Proteasome” pathway (KEGG database annotation) with high confidence.

### 3.3. The Workflow of Transcriptome- and Proteome-Based Regulatory Networks Design

The lists of DEGs and DEPs given in Supplemental Tables S2 and S5 have been used as the test sets (Yes-sets). The control sets were formed from the transcripts and proteins with unaltered expression as described in “Materials and Methods”. We performed the two-step bioinformatic analysis including:

1. Identification of TFs that can regulate the DEGs (MCD group) and DEPs at different time points after ATRA treatment using TRANSFAC@ database followed by matching putative TFs with the list of all transcripts identified (Supplemental Table S1) to cut-off the molecules that are not expressed in HL-60 cells at the mRNA level;
2. The upstream prediction of key molecules that regulate the TFs determined at the previous step using TRANSFAC@ database followed by visualization of the predicted interaction as a model regulatory networks.

To verify the molecules that are actually expressed in HL-60 cells, we matched the list of all identified and differentially expressed genes (Supplemental Table S1) and/or proteins (Supplemental Table S5) with the elements of model regulatory networks.

#### 3.3.1. The Transcriptome-Based Modeling Pathway

To find TFs responsible for regulation of gene expression we performed a search for the DEGs (MCD group) transcription factors binding sites (TFBS) at each time point studied (see results in Supplemental Table S6). TFs of DEGs determined at the 3/24 h and 24/96 h time were the same in general. So, in the case of time points 3, 24, and 96 h, we have combined all putative TFs in one set in order to perform key regulator search. The upstream analysis of the combined set of TFs, which are involved in regulation of MCD group genes at the 3 h, 24 h, and 96 h, revealed the top five key molecules with the lowest “Rank sum” value. The results are summarized in Table 1.

**Figure 4:**
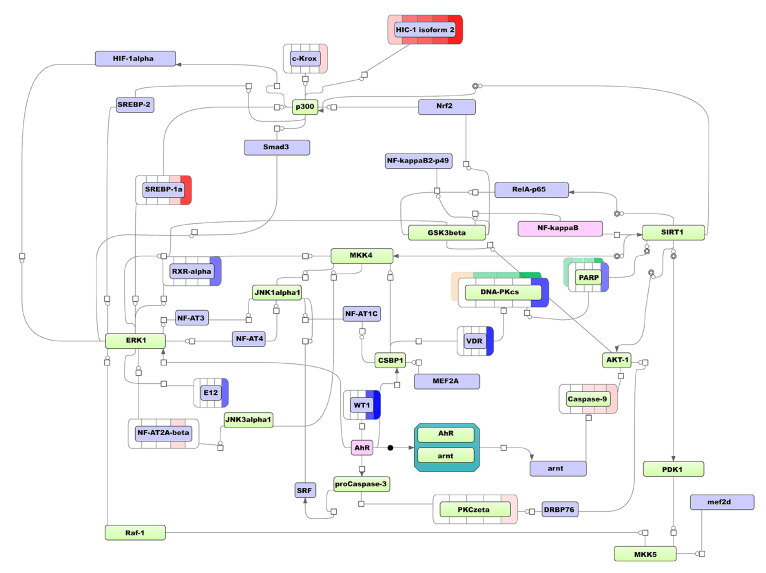
The transcriptome-based model network of regulation of MCD group DEGs during ATRA-induced HL-60 cells differentiation (the time points 3 h, 24 h, and 96 h). Legend: master regulatory molecules are represented by pink ellipses; connecting molecules considered by the graph-analyzing algorithm to find the path from the TF input list to the master molecule are represented by green ellipses; the molecules from the TF input list are represented by lilac ellipses. The colored bars around molecules show changes in the expression level. Transcript expressions are shown in blue (decreased expression) or pink (increased expression) color arrays, color intensity correlates with fold-change (FC), bars are colored if FC ≥ 2. From left to right each bar represent experimental time point (the time points at 3 h, 24 h, and 96 h and additional time points at 0.5 h and 1 h). Protein expression is shown in yellow (decreased expression) and green (increased expression) color array, color intensity correlates with fold-change (FC) of relative protein expression, bar is colored if FC ≥ 1.5, from left to right each bar represent experimental time point (3 h, 24 h, 48 h, and 96 h).

Further, to select the key molecules for visualization, we checked either its expressions were altered at ATRA-induced granulocytic differentiation (of primary importance), and compared FDR statistics. None of the key molecules from Table 1 were significantly changed at the transcript or protein levels. At the same time, AhR and NF-kappaB1 were the most reliable based on FDR value. Moreover, AhR and NF-kappaB1 mutually regulate each other according TRANSFAC@ database. The regulatory network triggered by AhR and NF-kappaB1 is shown in Figure 4.

According to the scheme, the key molecule AhR, apparently, causes down-regulation of proto-oncogene WT1, nuclear receptor RXRα, and transcription factor E12 (TCF3) and up-regulation of PKC zeta. AhR affects GSK3beta that regulates another key molecule, NF-kappaB1. On the other hand, NF-kappaB1 affects SIRT1 deacetylase, which inhibits the transcriptional activity of RelA/p65. NF-kappaB1 also influences GSK3beta kinase, thus performing the feedback and cross-regulation from two key molecules.

The model network also shows, that the NF-kappaB1/SIRT1 tandem down-regulates PARP1 (2-fold mRNA decrease at 96 h), DNA-PKcs (3-fold mRNA decrease at 96 h), and VDR (5-fold mRNA decrease at 96 h). VDR gene have been also indirectly controlled (via CSBP1) by AhR. Furthermore, NF-kappaB1/SIRT1 up-regulates TFs c-Krox, SREBP-1a, NF-AT2A-beta, and HIC1 mRNA expression. Both NF-kappaB1 and AhR trigger the up-regulation of caspase 9. These results indicate the synergistic effect of key molecules. Notably, transcriptome-based MCD-regulating scheme included various protein kinases (ERK, JNKalpha1, MKK4, GSK3beta, CSBP1 (MK14), AKT1, JNK3alpha1, Raf-1, PDK1, MKK5, and PKCzeta). This observation suggests the significant role of MAPK pathway in the regulation of DEGs of MCD group.

#### 3.3.2. The Proteome-Based Modeling Pathway

In the case of proteome data analysis, we have combined TFs which may regulate the expression of genes encoding DEPs (Supplementary Materials, Table S7). The results of the key regulator molecules search for DEPs are presented in Table 2. The Top-5 key molecules with the lowest “Rank sum” value are shown.

**Figure 5:**
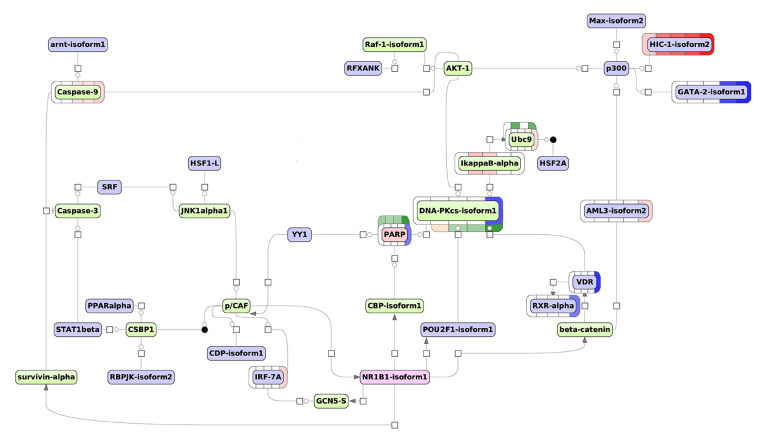
Proteome-based model network of regulation of ATRA-induced HL-60 cell line differentiation (time points 3 h, 24 h, 48 h, and 96 h). Legend: master regulatory molecules are represented by pink ellipses; connecting molecules considered by the graph-analyzing algorithm to find the path from the TF input list to the master molecule are represented by green ellipses; the molecules from the TF input list are represented by lilac ellipses. The colored bars around molecules show changes in the expression. Transcript expression is shown in blue (decreased expression) and pink (increased expression) color array, color intensity correlates with fold-change (FC) of relative mRNA expression, bar is colored if FC ≥ 2, from left to right each bar represent experimental time point (the time points at 3 h, 24 h, and 96 h and additional time points at 0.5 h and 1 h). Protein expression is shown in yellow (decreased expression) and green (increased expression) color array, color intensity correlates with fold-change (FC) of relative protein expression, bar is colored if FC ≥ 1.5, from left to right each bar represent experimental time point (3 h, 24 h, 48 h, and 96 h).

Further, to select the key molecule for visualization, we checked either its expression was altered during ATRA-induced granulocytic differentiation (of primary importance), and compared their FDR statistics. According to our transcriptomic data, we observed a 2-fold decrease of the PARP1 levels at 96 h. At the same time, PARP1 was identified in a shotgun mass spectrometry experiment. Furthermore, this molecule represents an intermediate node in the SIRT1-mediated signal transduction in the transcriptome-based network triggered by NF-kappaB1 and AhR (see Figure 4). In addition to the five most statistically significant molecular regulators, Table 2 also includes a retinoic acid receptor NR1B1 (RARα) as the key molecule. Although the Rank sum has not included RARα in the top five molecules, it has sufficient Score, Z-Score, and FDR values. Moreover, RARα is the well-known target of retinoic acid, inducing the differentiation of HL-60 cells [29]. The proteome-based scheme of TF regulation based on the selected key molecules, PARP1 and RARα, is shown in Figure 5. This modeling pathway could demonstrate molecular synergy of PARP1 and RARα.

Figure 5 demonstrates that in addition to the TFs with altered expression described previously (VDR, RXRα, and HIC1) the unique TFs were predicted using the proteomic data, including IRF7 and AML3 (RUNX2) (2.6-fold mRNA increased at 96 h), and GATA2 (mRNA reduced by 3.6- and 6.5-fold at 24 h and 96 h, respectively).

According to Figure 5, DNA-PKcs also affects IkappaB-alpha (NFKBIA): its expression is 3.2- and 2.9-fold increased at the transcriptome level at the time point 3 h.

Notably, the key molecule RARα (NR1B1 on the scheme) regulates PARP1 through CBP acetylase. In turn, the PARP1-triggered network regulates RARα through the DNK-PKcs/AKT1/CASP9/CASP3/SRF/JNK1α1/pCAF loop. In the case of RAR-dependent transcription, it has been found that PARP1 functions as a co-regulator, which is required to switch the mediator complex in the active state and start the transcription [30].

The same pathway branch (PARP1/DNA-PKcs/VDR) and some TFs (HIC1 and RXRα) belong to both transcriptome and proteome-based model regulatory networks that suggests the importance of these molecules and actual involvement of the pathways in the regulation of ATRA-induced differentiation of HL-60 cells.

### 3.4. Verification of Protein Levels of HIC1, PARP1, CEBPB, and LYN During ATRA-Induced Differentiation by SRM Analysis

**Figure 6:**
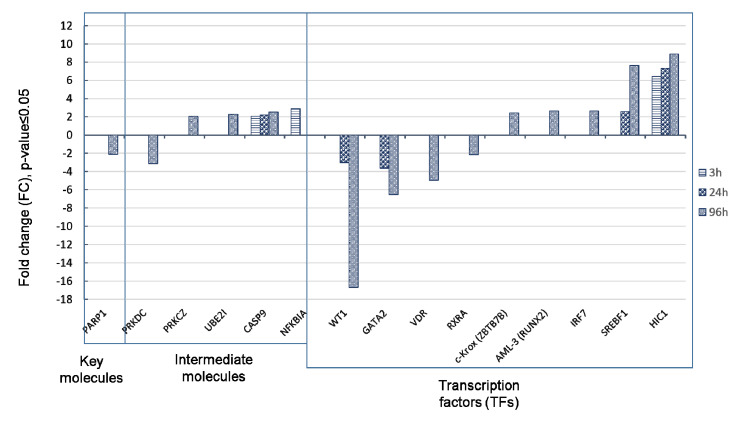
Components of transcriptome- and proteome-based model networks with altered mRNA expression under ATRA treatment. Transcription factors, TFs (predicted by TRANSFAC@ database), intermediate and key molecules (predicted by TRANSPATH@ database) with fold change ≥ 2 (*p*-value ≤ 0.05) at 3, 24, and 96 h is presented.

To reveal molecules of the transcriptome- and proteome-based pathways, which are actually expressed in HL-60 cells, we have matched the list of all identified and differentially expressed genes (Supplemental Table S1) and proteins (Supplemental Table S5) with molecules in the model regulatory networks. Differentially expressed genes belonging to the transcriptome- and proteome-based modeling networks are shown in Figure 6.

Figure 6 shows that 15 molecules, including one key molecule, five intermediate molecules, and nine transcription factors (TFs) of the transcriptome- and proteome-based model networks were characterized by the altered mRNA expression level. Transcriptional repressor HIC1 was strongly up-regulated at all time points studied suggesting its regulatory value. It is noteworthy that CASP9 and NFKBIA were up-regulated at 3 h after ATRA treatment. Transcription factors VDR and RXRA, which are intimately related to induced differentiation, were down-regulated (as well as key molecule PARP1).

**Figure 7:**
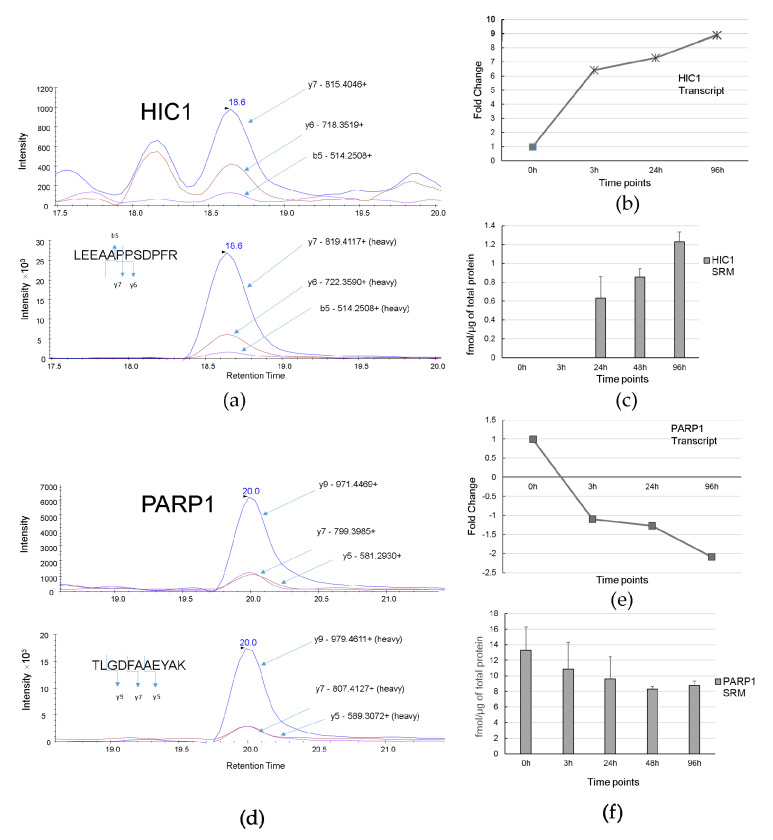
HIC1 and PARP1 expressions at ATRA-induced granulocytic differentiation. (**a**) Trace of SRM transitions for native and stable isotope labeled peptide standard LEEAAPPSDPFR of HIC1. (**b**) Profile of transcript expression HIC1 during HL60 differentiation (fold change ≥ 2, *p*-value ≤ 0.05 at 3 h, 24 h, and 96 h). (**c**) Protein expression level of HIC1 obtained by SRM (three biological replicates) at 3 h, 24 h, 48 h, 96 h. (**d**) Trace of SRM transitions for native and standard isotopically-labeled peptide TLGDFAAEYAK of PARP1. (**e**) Profile of transcript expression PARP1 during HL60 differentiation (fold change ≥ 2, *p*-value ≤ 0.05 at 96 h). (**f**) Protein expression level of PARP1 obtained by SRM (three biological replicates) at 3 h, 24 h, 48 h, 96 h.

**Figure 8:**
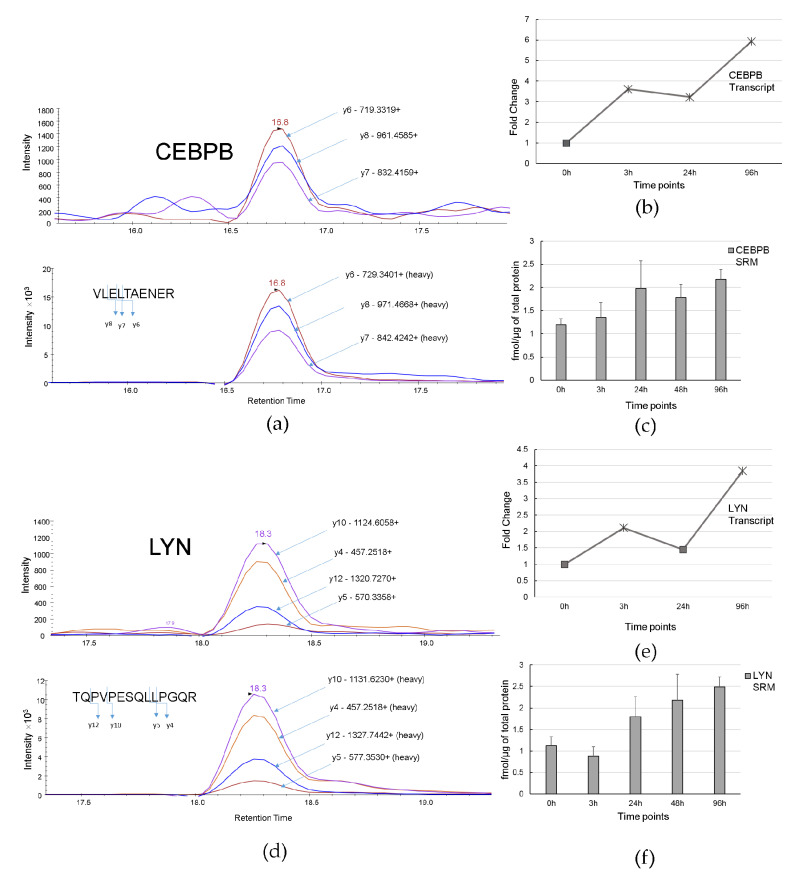
CEBPB and LYN expression during ATRA-induced granulocytic differentiation. (**a**) Trace of SRM transitions for native and stable isotope labeled peptide standard VLELTAENER of CEBPB. (**b**) Profile of CEBPB transcript expression during HL60 differentiation (fold change ≥ 2, *p*-value ≤ 0.05 at 3 h, 24 h, and 96 h) (**c**) Protein expression level of CEBPB obtained by SRM (three biological replicates) at 3 h, 24 h, 48 h, 96 h. (**d**) Trace of SRM transitions for native and standard isotopically-labeled peptide TQPVPESQLLPGQR of LYN. (**e**) Profile of transcript expression LYN during HL60 differentiation (fold change ≥ 2, *p*-value ≤ 0.05 at 3 h and 96 h). (**f**) Protein expression level of LYN obtained by SRM (three biological replicates) at 3 h, 24 h, 48 h, 96 h.

Among predicted regulatory molecules we selected transcription factor HIC1 and key molecule PARP1 for measuring abundance in HL-60 cells at different time points by SRM. Next, we have compared transcriptomic and proteomic profiles during ATRA-induced differentiation. We also evaluated levels of transcription factor CEBPB and LYN kinase with altered expression at the earliest time point (3 h) by SRM. Results are shown in Figure 7 and Figure 8.

Figure 7a,d demonstrate the trace of SRM transitions for native (above) and SIS standard (below) peptides LEEAAPPSDPFR of HIC1 protein, and TLGDFAAEYAK of PARP1 protein, respectively. The Figure 7b,c show transcriptomic and proteomic profiles of HIC1 expression. Transcription repressor HIC1 was up-regulated at 3 h and its mRNA abundance gradually increased almost 9 times to 96 h. HIC1 protein has not been identified in shotgun mass-spectrometry experiment. Using SRM technique with stable isotope labeled peptide standard (LEEAAPPSDPFR) the HIC1 abundance was detected at 24 h, 48 h, and 96 h. At these time-points its concentration was 0.63 ± 0.21 fmol/µg, 0.85 ± 0.14 fmol/µg, and 1.2 ± 0.15 fmol/µg of total protein, respectively. The HIC1 protein level was increased approximately 2-fold (FC = 1.9, *p*-value ≤ 0.05) from 24 h to 96 h after ATRA treatment.

The Figure 7e,f show transcriptomic and proteomic profiles of PARP1 expression. PARP1 was selected as a key molecule for the proteome-based model network. At the transcriptome level we revealed a 2-fold decrease in PARP1 mRNA expression at 96 h. The SRM measurements for the TLGDFAAEYAK peptide of PARP1 were 13.28 ± 2.98, 10.83 ± 3.46 fmol/µg, 9.57 ± 2.88 fmol/µg, 8.28 ± 0.35 fmol/µg, and 8.77 ± 0.54 fmol/µg of total protein at 0, 3 h, 24 h, 48 h, and 96 h after ATRA-treatment, respectively. The PARP1 protein level was 1.5-fold (*p*-value ≤ 0.05) down-regulated by 96 h after ATRA treatment.

Figure 8a,d demonstrate the trace of SRM transitions for native (above) and SIS standard (below) peptides VLELTAENER of CEBPB protein, and TQPVPESQLLPGQR of LYN protein, respectively.

Figure 8b demonstrates that CEBPB was up-regulated starting from 3 h (FC = 3.6, *p*-value ≤ 0.05) up to 96 h (FC = 5.95, *p*-value ≤ 0.05) at transcriptome level. Using SRM, we measured CEBPB in amount of 1.2 ± 0.12 fmol/µg, 1.36 ± 0.31 fmol/µg, 1.98 ± 0.59 fmol/µg, 1.78 ± 0.28 fmol/µg, and 2.17 ± 0.21 fmol/µg at 0, 3 h, 24 h, 48 h, and 96 h after ATRA-treatment, respectively (Figure 8c).

Figure 8e,f show transcriptomic and proteomic profiles of expression of LYN kinase. Transcriptomic data demonstrates significant LYN up-regulation at 3 and 96 h. The unique peptide (TQPVPESQLLPGQR, 21-34aa), which has been used for SRM analysis, is the LYN isoform B-specific and is mapped to the region that distinguishes isoform A from isoform B. High-resolution annotated MS2 spectrum of LYN isoform B-specific peptide TQPVPESQLLPGQR is shown in Figure S5. Protein LYN expression was detected in amount of 1.12 ± 0.2 fmol/µg, 0.8 ± 0.21 fmol/µg, 1.8 ± 0.46 fmol/µg, 2.18 ± 0.6 fmol/µg, and 2.49 ± 0.23 fmol/µg of total protein at 0, 3 h, 24 h, 48 h, and 96 h after ATRA-treatment, respectively.

We observed coordinate increase or decrease at the transcript and protein level for HIC1, CEBPB, LYN, and PARP1; this confirms involvement of corresponding genes in the ATRA induced HL60 differentiation. The targeted mass-spectrometric data have been uploaded into PASSEL repository (dataset PASS01678).

## 4. Discussion

Omics techniques provide a massive amount of data on the molecular state of the biological object studied. Nevertheless, in high-throughput transcriptome and proteome profiling, we always register only certain molecular consequences of regulatory events that occurred in the past (e.g., induction of the expression of the corresponding gene). Especially, proteomic research of differentiation onset is complicated by the fact that observed changes in protein levels take time. Thus, up-stream regulator search provides bioinformatics reconstruction of the molecular events up to one or several trigger points. Consistent with this, our whole-genome transcriptome results indicated activation of myeloid differentiation, whereas proteomic data demonstrated the involvement of the apoptosis pathways under ATRA treatment. However, knowing the expression differences alone does not allow us to reveal the effector that leads a biological system towards the particular molecular state. Applying up-stream regulator search and visualizing its result, we provide the putative “molecular scenarios” of how a dozen regulatory molecules decided the fate of hundreds of proteins and transcripts.

After ATRA treatment leukemic cells, of which the phenotype is generally driven by genetic abnormalities, acquire features of mature granulocytes. As in the case of many others malignancy, HL-60 cells harbor genetic aberrations including the most frequent mutations: extensive deletion of the *p53* gene, amplification of *MYC* oncogene, and monoallelic deletion of granulocyte–macrophage colony stimulating factor (*GM-CSF*) [11,12]. Considering this, we suggest that our model regulatory networks represent a putative way to overcome the effect of these mutations.

Proto-oncogene *MYC* plays a crucial role in the regulation of cell proliferation, differentiation, and apoptosis [31,32]. From 16- to 32-fold *MYC* gene amplification in the HL-60 genome has been reported [33]. Although the decreased expression of *MYC* is not sufficient for triggering differentiation of HL-60 cells, it is accompanied by the inhibition of cell growth [34]. In our study, we observed a 7-fold decrease of MYC mRNA expression during granulocytic differentiation. Notably, TF MAX that binds MYC protein for activation of target genes [35] is the part of our proteome-based model network. Thus, the modeling scheme presented in Figure 4 could represent a way to overcome the deleterious effect of MYC gene amplification.

Normally, the *p53* gene is a crucial component of the molecular response to different kinds of cell stress including DNA damage. Namely, p53 is involved in mismatch repair, DNA double-strand break repair, and nucleotide excision repair that could accompany uncontrolled proliferation [36]. Poly(ADP-ribose) polymerase 1 (PARP1), the key molecule of the proteome-based model network, has intricate interplay with p53 in regulation of cell death and survival. PARP1 affects p53 transcriptional activity, and promotes its oncosupressive function [37]. In turn, the p53 expression level is prominently increased after DNA damage in PARP1-defiecint cells that leads to apoptosis [38]. Moreover, in the case of the multidrug-resistant leukemia cell line HL-60[R] the PARP1 mRNA expression level was up-regulated [39]. At the same time, a branch of components PARP1/DNA-PKcs/VDR, which is presented both the in transcriptome- and proteome-based model pathways (Figure 4 and Figure 5), regulates DNA repair [40,41]. Thus, the proteome-based model network could represent a molecular bypass to overcome consequences of *p53* deletion. It may be assumed that inhibition of PARP1 in p53-deficient HL-60 cells could have the similar antiproliferative effect as on BRCA1-deficient cancer cells of solid tumors [42]. This assumption is in agreement with the fact that primary blasts from patients with acute myeloid leukemia are sensitive to PARP-inhibitor Olaparib [43].

In our study SRM measurements show a trend of the diminution of PARP1 protein abundance, while the mRNA level was significantly down-regulated, 2-fold, to 96 h after ATRA-treatment. Considering the moderate modulation of abundance it is conceivable that PARP1 is regulated by post-translation modification. Figure 4 and Figure 5 demonstrate that PARP1 could be acetylated by CREB-binding protein (CBP) or deacetylate by SIRT1. Both PARP1 and SIRT1 compete for the common NAD+ substrate and modulate each other’s activity by mutual modification [44]. PARP1 inhibition by SIRT1 could contribute to the increase in the DNA damage level and cell death in the absence of *p53* expression. SIRT1 stimulation by pharmacological agents could promote PARP1 inhibition. On other hand, SIRT1 can activate apoptosis by direct deacetylation of the RelA-p65 subunit that inhibits the transcription of NF-kappaB and increases cell sensitivity to TNF-alpha-induced apoptosis [40]. TNF-alpha is known to cause p53-independent apoptosis, which promotes the monocytic differentiation of HL-60 cells [45].

At the same time, we observed prominent up-regulation of transcriptional repressor HIC1 that suppresses SIRT1 gene expression. SIRT1 deacetylates and inactivates both p53 and PARP1; HIC1 affects cell cycle, apoptosis, and DNA repair. According to our transcriptome-based model network (Figure 4), HIC1 was triggered by NF-kappaB via SIRT1 and p300. In Figure 5, a proteome-based model network represents HIC1 regulated by cascade triggered by PARP1 through DNA-PKs, AKT, and p300. This suggests a feedback loop involved in maintaining moderate inhibition of SIRT1 via HIC1 that sustains PARP1 activity, resulting in delayed apoptosis and allowing cells to differentiate into neutrophils. Apparently, accumulation of critical amount of HIC1 causes SIRT1 suppression, and further PARP1 down-regulation occurs due to apoptosis-driven cleavage. It seems that the cell machinery involved in the response to the DNA damage plays a key role in induced granulocytic differentiation, and its component could be sensitive to target treatment.

The transcriptome analysis provides biological data on ATRA-induced granulocytic differentiation at the whole genome-scale. However, not all transcripts detected could be traced at the protein level. In turn, despite the proteomic data being limited by the sensitivity of mass-spectrometry, the protein expression underlies the cell phenotype manifestation. As expected, different inputs to up-stream regulator search resulted in different key molecules in transcriptome- and proteome-based modeling pathways. Still, the schemas show common predicted transcription factors (SRF, ARNT, RXRA, VDR, and HIC1), intermediate molecules (Caspase9, histone acetyltransferase p300, protein kinases ERK1, Raf-1, AKT1, CSBP1 (MK14), JNKaplha1, and AKT), and even whole branches of molecular events (axis PARP1-DNA-PKcs-VDR). The gene transcription and protein synthesis are separated in time, and the above observations suggest different key regulation, but we also observe the general molecular consequences, such as the involvement of the DNA repair system and the MAPK kinase cascade.

Interesting but conflicting results were obtained for LYN kinase. The previous studied demonstrated that constitutively activated LYN was involved in AML pathogenesis and treatment of cells by LYN siRNA resulted in the antiproliferative effect [46,47]. In our study we observed LYN up-regulation at mRNA level under ATRA treatment. SRM technique allows to distinguish different isoforms of the same protein. We used the isoform specific peptide standard to detect LYN isoform B and found it to be up-regulated at the proteome level. Previously it was reported that phosphorylation activity of Lyn isoform B was lower than that of Lyn isoform A [48]. Moreover, the ratio of Lyn isoform A and Lyn isoform B splice forms may represent a biomarker of neoplasm aggressiveness as was shown in the case of breast cancer [49].

Absolute quantification by SRM with SIS peptides demonstrates the almost equimolar abundance of TF CEBPB and Src kinase LYN. Considering their possible interaction (STRING analysis of DEGs, Figure S2), absolute abundances of CEBPB and LYN suggest protein stoichiometry in the putative complex involved in the earliest step of ATRA-induced granulocytic differentiation.

The myeloid-associated TFs (RARa, RXR, VDR, CEBPB, and GATA2) of model schemes confirm the biological relevance of bioinformatics modeling. Notably, a transcriptome-based MCD-regulating scheme included various protein kinases (ERK, JNKalpha1, MKK4, GSK3beta, CSBP1 (MK14), AKT1, JNK3alpha1, Raf-1, PDK1, MKK5, and PKCzeta), that is in accordance with MAPK-based mechanisms for ATRA-induced granulocytic differentiation [14]. Moreover, the current inter-platform study shows the involvement of such less associated with AML TFs as NF-ATs, SMAD3, WT1, and c-Krox, as well as ubiquitous molecules (p300, P/CAF, UBC9), which are involved in posttranslational modifications (acetylation, sumoylation, ubiqutunilation etc.). All the above observations suggest the existence of alternative, RAR/RXR transcription-independent, induced differentiation pathways. However, this assumption should be experimentally proven.

## 5. Conclusions

Applying transcriptomic, proteomic analysis, and bioinformatics prediction we have suggested a hypothesis on molecular mechanism of ATRA-induced granulocytic differentiation. We aimed to trace dynamics at different molecular levels in a time-course manner. The novelty of the approach used in our study is that molecules with altered expression from omics experiments have not been just mapped to known signaling pathways. Instead, an upstream regulator search aimed to obtain the hierarchical model of ATRA-induced granulocytic differentiation that reconstructs the molecular events affecting differentially expressed mRNA and proteins. Only the TFBS in the promotor region of genes with altered expression and highly validated data on protein–protein interaction were taken into account in upstream regulator search. The resulting modeling schemas are visualizations of the most probable variant of a biological signal transmission, which leads to a change in the expression levels of transcripts and proteins, observed experimentally. The validation of bioinformatics prediction by functional molecular research is an important item, and a subject of our further work. The TF HIC1 and the key molecule PARP1 are contemplated as the most promising targets for validation of the modeling pathways.

The approach combining transcriptomic, proteomic analysis, and computational analysis described here is applicable to various cells models including primary blast cells from patients under different treatment regimens. Thus this platform could be useful for the goals of precision medicine such as monitoring response to treatment especially in case of drug resistance. Our results suggest that the multi-disciplinary platform combining transcriptomics, proteomics, and bioinformatics is a promising approach to reveal regulatory molecules that are hardly detected by convenient omics methods or laborious to derive from convoluted proteomic or transcriptomic data.

## Figures and Tables

**Table 1:** Putative key molecules responsible for regulation of the DEGs related to the myeloid cell differentiation (MCD group) at 3, 24 and 96 h after ATRA treatment.

Time Point.	Key Molecule Name	Reached from TF Set ^1^	Reachable Total ^2^	Score ^3^	FDR ^4^	Z-Score ^5^	Ranks Sum ^6^
3-24-96 h (MCD)	AhR	22	12488	0.34	0.011	2.31	5
	arnt	21	8990	0.34	0.026	2.1	6
	Nrf2	15	9200	0.24	0.033	2.61	6
	(CKII-α)2:(CKII-β)2	22	10803	0.28	0.025	2.45	8
	NF-kappaB1	21	10897	0.29	0.024	2.05	11

^1^ “Reached from TF set”—the number of the TFs from the input set (Supplemental Table S6) that is reached from the respective key molecule; ^2^ “Reachable total”—the total number of molecules that can be reached from the key molecule, independent of the input set; ^3^ “Score”—the value reflecting how well the respective key molecule is connected with other molecules in the database, and how many molecules from the input set are present in the network triggered by this key molecule, the higher value—the better suitability (threshold value > 0.2); ^4^ FDR—false discovery rate (from 1000 random input sets); ^5^ “Z-score”—the value that reflects how specific each key molecule is for the input list, the higher value—the better suitability (threshold value > 1); ^6^ “Rank sum”—composite value that reflects the impact of Score and Z-score simultaneously, the lower value—the better suitability.

**Table 2:** Putative key molecules that regulate DEPs at 3, 24, 48, and 96 h during ATRA-induced differentiation of HL-60 cells.

Time Point	Key Molecule Name	Reached from TF Set ^1^	Reachable Total ^2^	Score ^3^	FDR ^4^	Z-Score ^5^	Ranks Sum ^6^
Combined 3-24-48-96 h	YY1	22	32835	0.650	0.013	2.779	59
	plk1{p}	22	32762	0.640	0.002	2.803	63
	PARP1	22	32360	0.607	0.006	2.950	63
	faim	22	32361	0.607	0.006	2.950	64
	MKK6	22	33716	0.721	0.004	2.325	88
	NR1B1 (RARA)	22	30223	0.505	0.018	3.007	204

^1^ “Reached from TF set”—the number of the TFs from the input set (Supplemental Table S7) that is reached from the respective key molecule; ^2^ “Reachable total”—the total number of molecules that can be reached from the key molecule, independent of the input set; ^3^ “Score”—the value reflecting how well the respective key molecule is connected with other molecules in the database, and how many molecules from the input set are present in the network triggered by this key molecule, the higher value—the better suitability (threshold value > 0.2); ^4^ FDR—false discovery rate (from 1000 random input sets); ^5^ “Z-score”—the value that reflects how specific each key molecule is for the input list, the higher value—the better suitability (threshold value > 1); ^6^ “Rank sum”—composite value that reflects the impact of Score and Z-score simultaneously, the lower value—the better suitability.

## Data Availability

Mass-spectrometric data are available via ProteomeXchange with identifier PXD006768.

## Supplementary Materials

The following are available online at https://www.mdpi.com/article/10.3390/biom11060907/s1, Figure S1. Evaluation of HL-60 cell line by the CD38 and CD11b expression level measured by flow cytometry. Figure S2. STRING interaction analysis of differentially expressed genes (DEGs) of Myeloid cell differentiation (MCD) group at 3, 24, and 96 h after ATRA treatment. Figure S3. Heatmap of protein expression during HL-60 cell line differentiation. Figure S4. STRING interaction analysis of differentially expressed proteins (DEPs) assigned to group “programmed cell death” and/or “regulation of cell death” at 3 and 96 h after ATRA treatment. Figure S5. High-resolution annotated MS2 spectrum of LYN isoform B-specific peptide TQPVPESQLLPGQR. Figure S6. Calibration curves plotting of experimentally determined concentrations versus theoretical concentrations of the target analyte using isotopically labeled and label-free synthetic standard peptide A: LEEAAPPSDPFR (HIC1), B: TLGDFAAEYAK (PARP1), C: VLELTAENER (CEBPB), and D: TQPVPESQLLPGQR (LYN). Table S1. All transcripts detected and differentially expressed genes (DEGs) with fold-change equal or above 2 (*p*-value < 0.05) at 3 h, 24 h, and 96 h after ATRA treatment. Table S2. The transcriptomic test sets (Yes-sets) for pathway modeling: DEGs related to the myeloid cell differentiation (MCD) (GO: 0030099) at the 3, 24, and 96 h time points. Table S3. Data on spectral counting. Number of unique peptides, percent of coverage, number of spectra, q-values and local FDR are shown for each peptide in each time point (0, 3, 24, 48, and 96 h). Table S4. Data on spectral counting. Number of unique proteins, percent of coverage, number of spectra, q-values and local FDR are shown for each protein in each time point (0, 3, 24, 48, and 96 h). Table S5. Summary of relative expression analysis and proteomic test sets (Yes-sets) for pathway modeling. Table S6. Transcription factors (TFs) possibly regulating DEGs expression during ATRA-induced HL-60 cell differentiation at time points 3 h, 24 h, and 96 h. Table S7. Transcription factors (TFs) possibly regulating DEPs expression during ATRA-induced HL-60 cell differentiation at time points 3 h, 24 h, 48 h, and 96 h. Table S8. Transition for SRM method (QqQ TSQ Vantage (Thermo Scientific, USA).
