# Supplementary material for: Omics Technologies to Decipher Regulatory Networks in Granulocytic Cell Differentiation
Source: Biomolecules. 2021 Jun 18;11(6):907. doi: 10.3390/biom11060907 (PMC8233756; doi:10.3390/biom11060907)
Supplement: Supplementary file 1 [file biomolecules-11-00907-s001.zip › biomolecules-1214844-supplementary.pdf]

biomolecules

Supplementary materials

Omics Technologies to Decipher Regulatory Networks in Granulocytic Cell Differentiation

Svetlana Novikova †, Olga Tikhonova †, Leonid Kurbatov, Tatiana Farafonova, Igor Vakhrushev, Alexey Lupatov, Konstantin Yarygin and Victor Zgoda *

Orekhovich Institute of Biomedical Chemistry, Pogodinskaya 10, Moscow 119121, Russia; novikova.s.e3101@gmail.com (S.N.); ovt.facility@gmail.com (O.T.); kurbatovl@mail.ru (L.K.); farafonova.tatiana@gmail.com (T.F.); vakhrunya@gmail.com (I.V.); alupatov@inbox.ru (A.L.); kyarygin@yandex.ru (K.Y.); victor.zgoda@gmail.com (V.Z.)

* Correspondence: victor.zgoda@gmail.com

† These authors contributed equally to this work

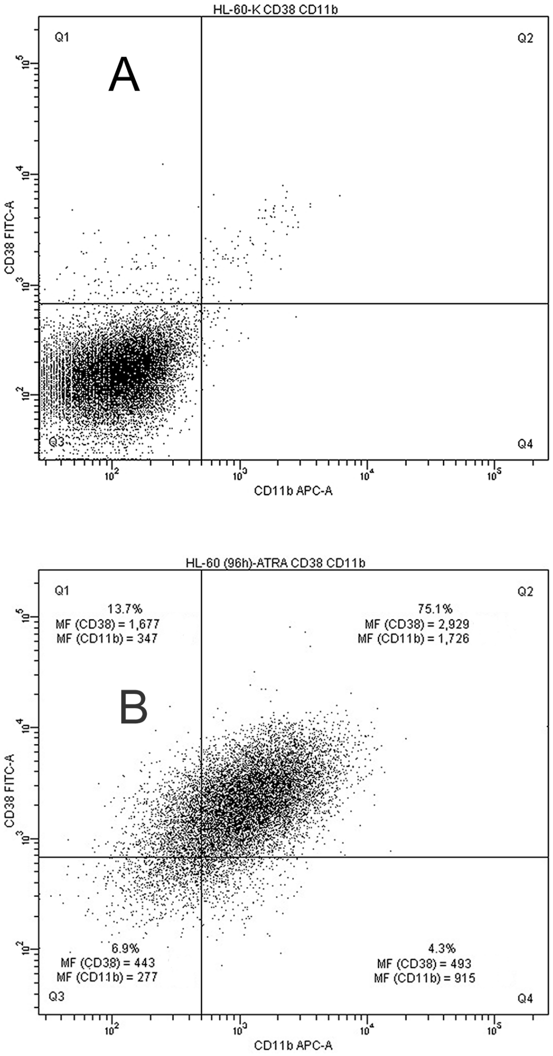

Figure S1. Evaluation of HL-60 cell line by the CD38 and CD11b expression level measured by flow cytometry: (a) untreated cells and (b) cells at 96 h after ATRA treatment.

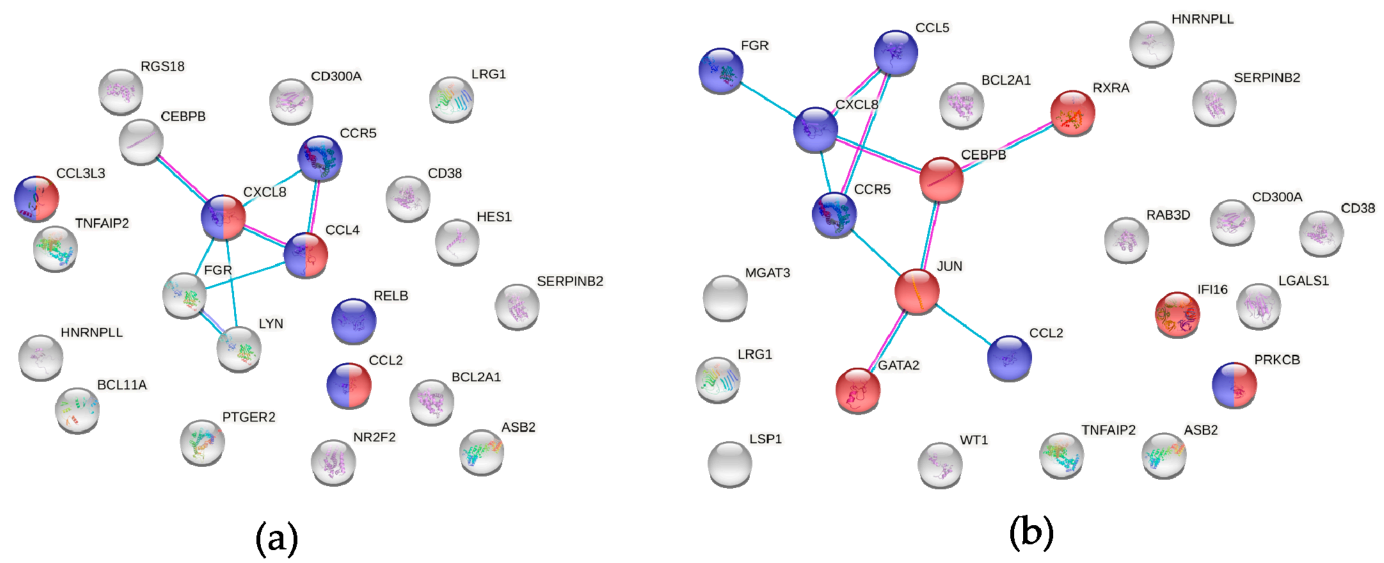

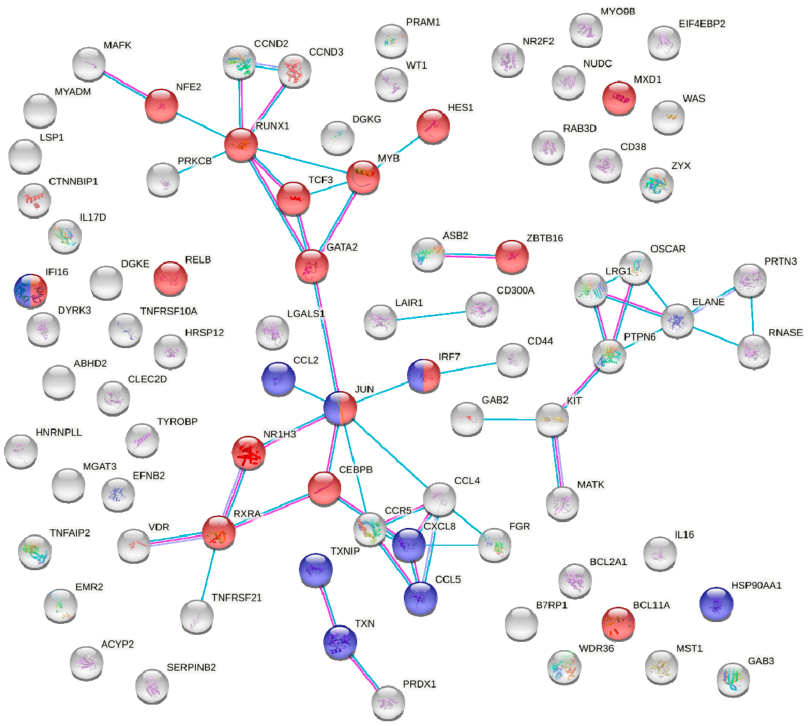

(c)

Figure S2. STRING interaction analysis (a) for 22 differentially expressed genes (DEGs) of MCD group (3h) enriched in interactions (PPI enrichment p-value = 0.00595), red and blue nodes shows DEGs (MCD group) that have been annotated by Molecular function (GO) as belonging to "chemokine activity" group (FDR = 5.70e-05) and by KEGG database as members of "Chemokine signaling pathway" group (FDR = 6.38e-08), respectively; (b) for 24 DEGs (MCD group, 24 h) enriched in interactions (PPI enrichment p-value = 0.00684), red and blue nodes shows DEGs (MCD group) that have been annotated by Molecular function (GO) as belonging to "transcription factor binding" group (FDR = 0.0055) and by KEGG database as members of "Chemokine signaling pathway" group (FDR = 9.86e-06), respectively; (c) for 81 DEGs (MCD group, 96 h) enriched in interactions (PPI enrichment p-value = 5.31e-07), red and blue nodes shows DEGs (MCD group) that have been annotated by Molecular function (GO) as belonging to "proximal promoter sequence-specific DNA binding" group (FDR = 1.08e-07) and by KEGG database as members of "NOD-like receptor signaling pathway" group (FDR = 3.39e-06), respectively.All networks were enriched using the intersection of 8612 genes present on all platforms as the background, along with evidence from experimental protein–protein interactions (PPI) (purple lines), and curated (turquoise blue lines) databases. The network was built based on the highest confidence (0.9).

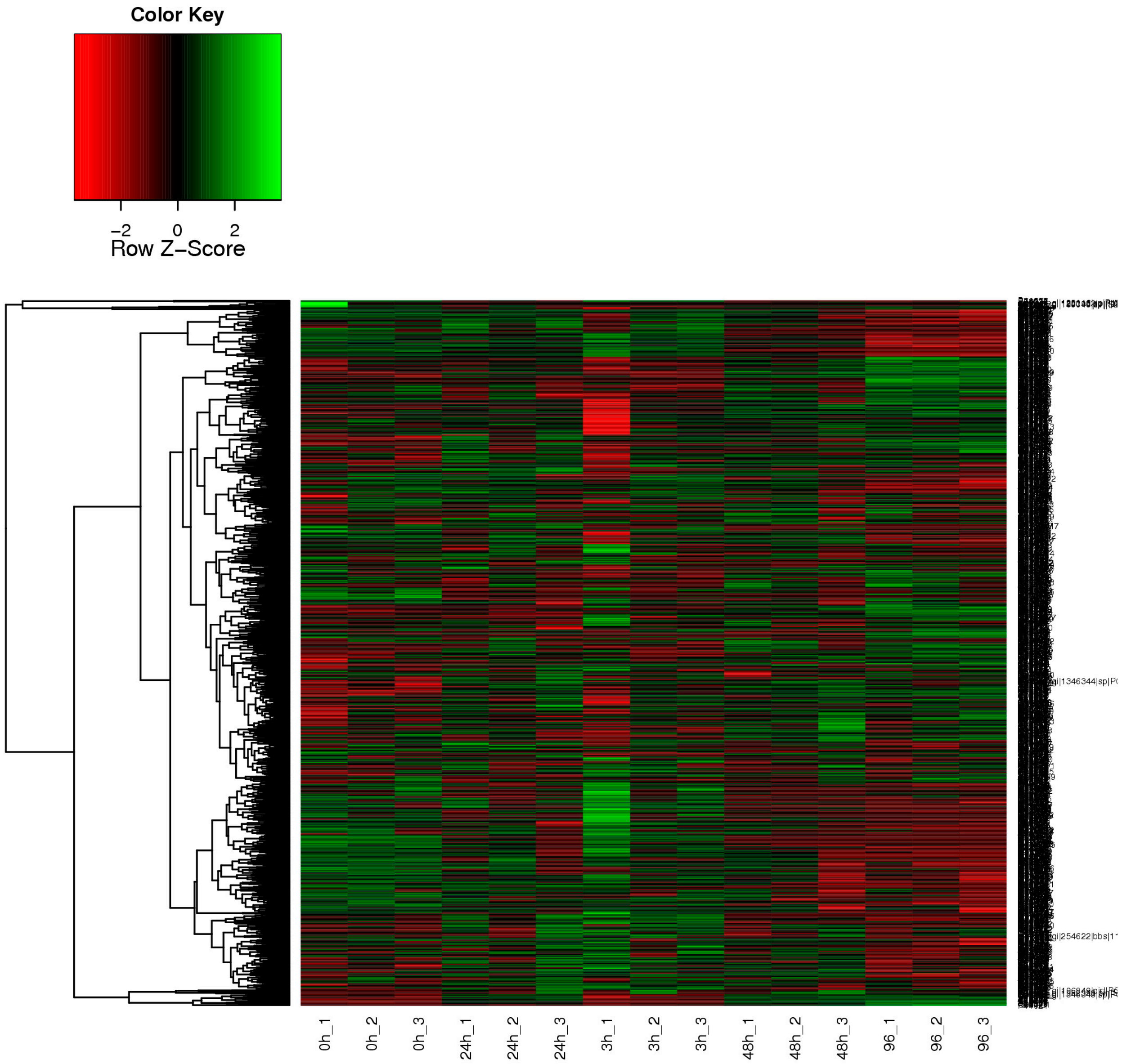

Figure S3. Heatmap of protein expression. Expression color coded from low (dark red) to high (dark green). The figure reflects clusterization of proteins identified at 0, 3, 24, 48 and 96 h after ATRA treatment in 3 biological replicates.

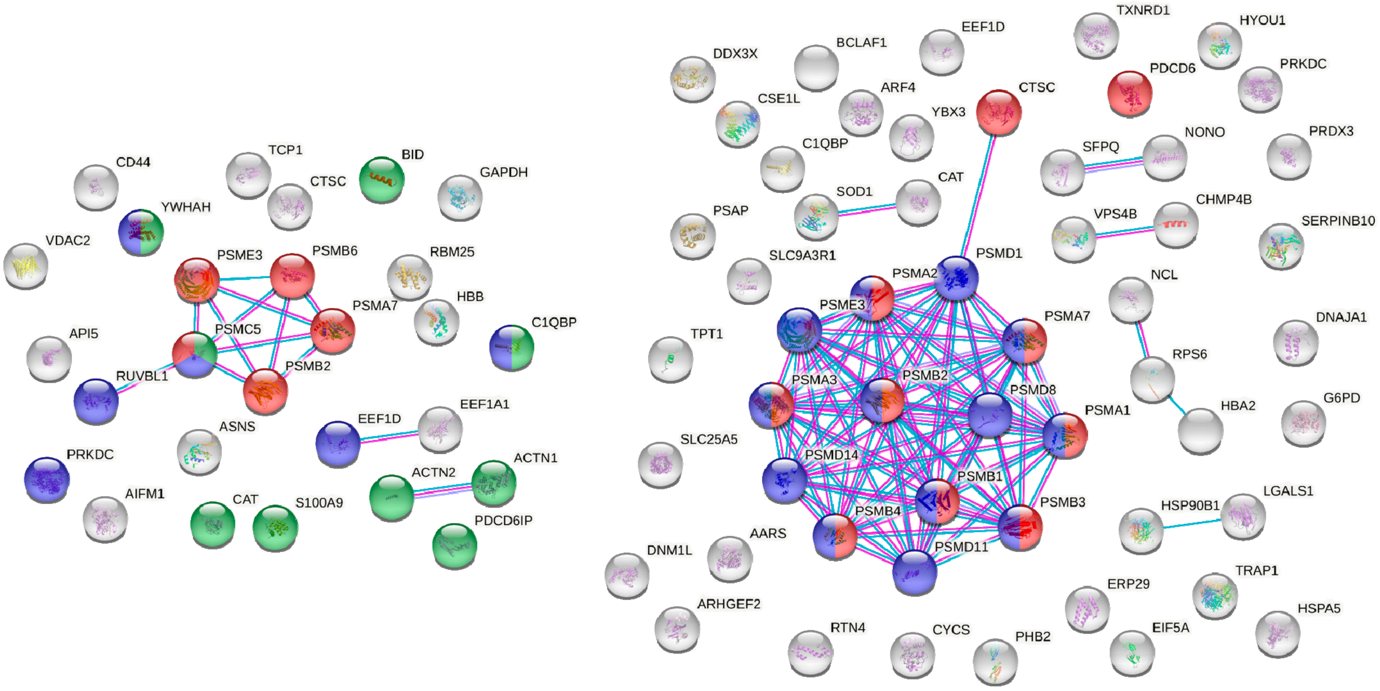

(a) (b)

Figure S4. STRING interaction analysis of differentially expressed proteins (DEPs) assigned to group "programmed cell death" and/or "regulation of cell death" at 3 and 96 h after ATRA treatment;(a) 27 DEPs at 3 h after ATRA treatment enriched in interactions (PPI enrichment p-value = 0.000248), red, blue, and green nodes shows DEPs that have been annotated by Molecular function (GO) as belonging to "signaling receptor binding" group (FDR = 0.0045), and to "transcription factor binding" group (FDR = 0.0048), as well as by KEGG database as members of "Proteasome" pathway (FDR = 3.75e-07), respectively; (b) 54 DEPs at 96 h after ATRA treatment enriched in interactions (PPI enrichment p-value = 1.0e-16), red and blue nodes shows DEPs that have been annotated by Molecular function (GO) as belonging to "endopeptidase activity" group (FDR = 9.44e-06) and by KEGG database as members of "Proteasome" pathway (FDR = 1.38e-20), respectively.

Table S6. Transcription factors (TFs) that are responsible for the change in gene expression of Myeloid Cells Differentiation (MCD) group MCD at time points 3, 24 and 96 hours in the course of differentiation of HL-60 cells. TFs found also as potential regulators of differentially expressed proteins (see Table S8, below) are shown in bold. TFs demonstrating altered expression during ATRA-induced differentiation of HL-60 cells are underlined.

TF gene name, 3 h	TF gene name, 24 h	TF gene name, 96 h
	AHR	AHR
	**ARNT**	**ARNT**
BACH1	BACH1	
		**HIC1**
	HIF1A	HIF1A
	**HSF2**	**HSF2**
ILF3	ILF3	
MAFB	MAFB	
MAFG	MAFG	
MAFK	MAFK	
		MAZ
MEF2A	MEF2A	
MEF2D	MEF2D	
NFATC1	NFATC1	
NFATC2	NFATC2	
NFATC3	NFATC3	
NFATC4	NFATC4	
NFE2	NFE2	
NFE2L1	NFE2L1	
NFE2L2	NFE2L2	
NFE2L3	NFE2L3	
	NFKB1	
	NFKB2	
	NKX2-5	
	RELA	

	**RXRA**	
	RXRB	
SMAD3	SMAD3	
SREBF1	SREBF1	
SREBF2	SREBF2	
**SRF**	**SRF**	**SRF**
TCF3		TCF3
		TFAP2C
		TFCP2
	**VDR**	**VDR**
	WT1	WT1
	ZBTB7A	ZBTB7A
		ZBTB7B (c-Krox)

Table S7. Transcription factors (TFs) possibly regulating protein expression during ATRA-induced HL-60 cell differentiation at time points 3 h, 24 h, 48 h, and 96 h. Common TF that also may be regulators of differentially expressed transcripts are shown in bold. TFs demonstrating altered expression during ATRA induced differentiation of HL-60 cells are underlined.

TF gene name, 3h	TF gene name,24h	TF gene name, 48h	TF gene name, 96h
**ARNT**	**ARNT**		
CUX1 (CDP)	CUX1 (CDP)		
	**EP300**		
			<u>GATA2</u>
	**<u>HIC-1</u>**		
	HSF1	HSF1	
		**HSF2**	**HSF2**
<u>IRF7</u>			
		LMO2	
			**MAX**
	NFYC	NFYC	NFYC
NKX31 (NKX3A)			NKX31 (NKX3A)
NR1H3			
PBX3	PBX3	PBX3	
POU2F1	POU2F1	POU2F1	
	**PPARA**	**PPARA**	
RARA (NR1B1)			
			RBPJ (RBPJK)
		RFXANK	
		<u>RUNX2 (AML3)</u>	
**<u>RXRA</u>**			
	**STAT1**	**STAT1**	**STAT1**
		**SRF**	
	**<u>VDR</u>**	**<u>VDR</u>**	**<u>VDR</u>**
	YY1		
ZNF384			

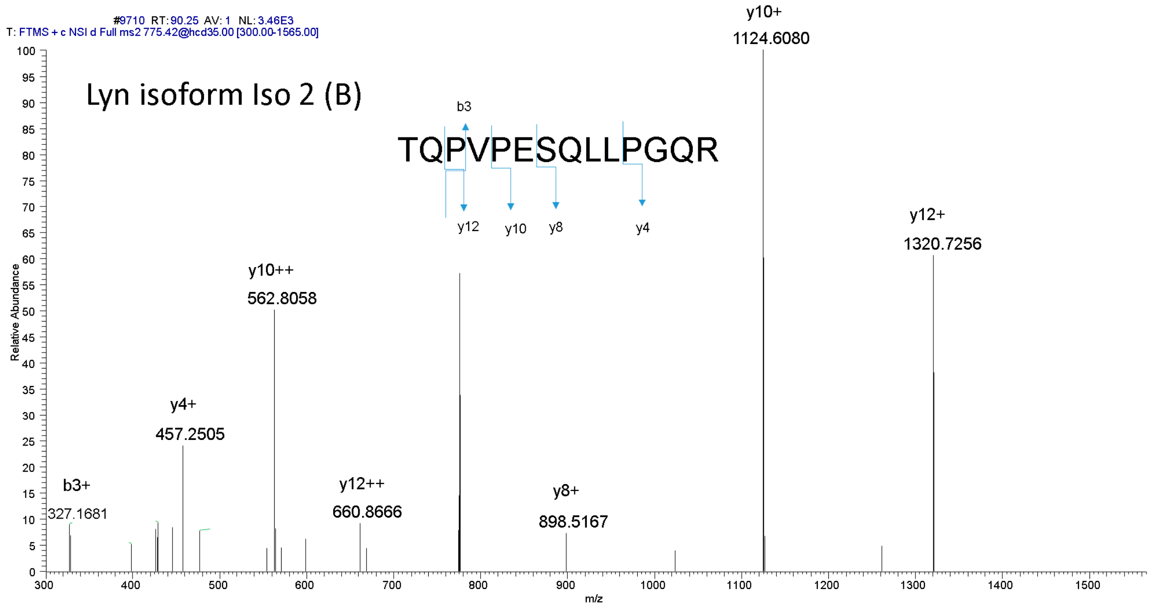

Figure S5. High-resolution annotated MS2 spectrum of LYN isoform B-specific peptide TQPVPESQLLPGQR.

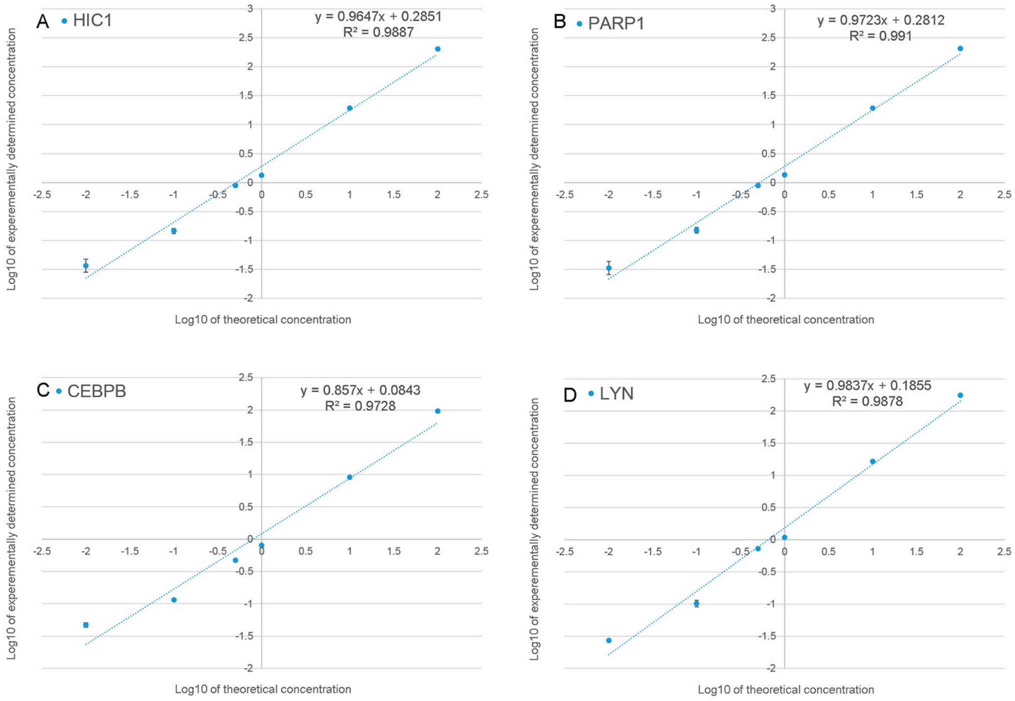

Figure S6. Calibration curves plotting of experimentally determined concentrations versus theoretical concentrations of the target analyte using isotopically labeled and label-free synthetic standard peptide (a) LEEAAPPSDPFR (HIC1), (b) TLGDFAAEYAK (PARP1), (c) VLELTAENER (CEBPB), and (d) TQPVPESQLLPGQR (LYN). Isotopically labeled synthetic peptide was added to concentration of 12.5 fmol/µl into serial dilutions (0.01, 0.1, 0.5, 1, 10, 100 fmol/µl) of label-free synthetic peptide. Calibration curve is presented in Log10 scale.

Table S8. Transition for SRM method (QqQ TSQ Vantage (Thermo Scientific, USA), isotopically-labeled amino acid is shown in bold. +—singly-charged ion; ++—doubly-charged ion

Peptide sequence	Precursor ion m/z	Fragment ion m/z	Collision energy
TLGDFAAEYAK	593.293^{++}	971.4469^{+}	20.7
		799.3985^{+}	
		581.293^{+}	
TLGDFAAEYA**K** (heavy)	597.3001^{++}	979.4611^{+}	
		807.4127^{+}	
		589.3072^{+}	
LEEAAPPSDPFR	664.8277^{++}	815.4046^{+}	22.8
		718.3519^{+}	
		514.2508^{+}	
LEEAAPP**S**DPFR (heavy)	666.8312^{++}	819.4117^{+}	
		722.359^{+}	
		514.2508^{+}	
VLELTAENER	587.3091^{++}	961.4585^{+}	20.5
		832.4159^{+}	
		719.3319^{+}	
VLELTAENE**R** (heavy)	592.3133^{++}	971.4668^{+}	
		842.4242^{+}	
		729.3401^{+}	
TQPVPESQLLPGQR	775.4203^{++}	1320.7270^{+}	26.2
		1124.6058^{+}	
		570.3358^{+}	
		457.2518^{+}	26.2
TQPVPESQL**L**PGQR (heavy)	778.9289^{++}	1327.7442^{+}	
		1131.6230^{+}	
		577.3530^{+}	
		457.2518^{+}	
